# 2025 Consensus Clinical Management Guidelines for Niemann‐Pick Disease Type C

**DOI:** 10.1002/jimd.70185

**Published:** 2026-04-26

**Authors:** Tarekegn Hiwot, Forbes D. Porter, Tatiana Bremova‐Ertl, Uma Ramaswami, Caroline Hastings, Bénédicte Héron, Justin Hopkin, Joella Melville, Hernan Amartino, Mireia del Toro, Federica Deodato, Fatih Ezgü, Paul Gissen, James B. Gibson, Can Ficicioglu, Roberto Giugliani, Orna Staretz‐Chacham, Frances Platt, Nathalie Guffon, Kristina Julich, Nikola Kresojević, Anna Lehman, Yann Nadjar, Susanne A. Schneider, Simon Jones, Eugen Mengel, Michel Tchan, Mark Walterfang, Ozlem Goker‐Alpan, Charlotte Dawson, Sandra Cowie, Toni Mathieson, Elizabeth Berry‐Kravis, Marc C. Patterson

**Affiliations:** ^1^ Institute of Metabolism and System Research University of Birmingham Birmingham UK; ^2^ Department of Endocrinology University Hospital Birmingham Birmingham UK; ^3^ Eunice Kennedy Shriver National Institute of Child Health and Human Development National Institutes of Health Bethesda Maryland USA; ^4^ Department of Neurology University Hospital Inselspital Bern Switzerland; ^5^ Royal Free London NHS Foundation Trust London UK; ^6^ University College London London UK; ^7^ UCSF Benioff Children's Hospital Oakland Oakland California USA; ^8^ Department of Pediatric Neurology, Reference Center of Lysosomal Diseases Armand Trousseau Hospital Paris France; ^9^ Hospitalo‐Universitary Federation I2‐D2 Sorbonne‐Université, Assistance Publique‐Hôpitaux de Paris Paris France; ^10^ University of Rochester School of Medicine and Dentistry Rochester New York USA; ^11^ 67health London UK; ^12^ Servicio de Neurología Infantil Hospital Universitario Austral and Instituto Neurogenia Buenos Aires Argentina; ^13^ Pediatric Neurology Department, Unit of Hereditary Metabolic Disorders Hospital Universitari Vall d'Hebron Barcelona Spain; ^14^ Division of Metabolic Diseases and Hepatology Ospedale Pediatrico Bambino Gesù, IRCCS Rome Italy; ^15^ Department of Pediatrics, Faculty of Medicine Gazi University Ankara Turkey; ^16^ Department of Inborn Metabolic Diseases, Faculty of Medicine Gazi University Ankara Turkey; ^17^ National Institute for Health Research, Great Ormond Street Hospital, Biomedical Research Centre University College London London UK; ^18^ Metabolic Genetics Dell Children's Medical Group Austin Texas USA; ^19^ Department of Pediatrics Dell Medical School at the University of Texas at Austin Austin Texas USA; ^20^ The Children's Hospital of Philadelphia, Division of Genetics/Metabolism Perelman School of Medicine at the University of Pennsylvania Philadelphia Pennsylvania USA; ^21^ Universidade Federal do Rio Grande do Sul, Hospital de Clinicas de Porto Alegre, IMASP, Dasa Genomica, Casa dos Raros Porto Alegre Brazil; ^22^ Center for Rare Diseases, Soroka Medical Center BenGurion University Beer Sheva Israel; ^23^ Department of Pharmacology University of Oxford Oxford UK; ^24^ Reference Center for Inborn Errors of Metabolism, Hôpital Femme Mère Enfant Hospices Civils de Lyon Bron France; ^25^ The University of Texas at Austin Dell Medical School Austin Texas USA; ^26^ Neurology Clinic, University Clinical Centre of Serbia, Faculty of Medicine University of Belgrade Belgrade Serbia; ^27^ Faculty of Medicine, Department of Medical Genetics University of British Columbia Vancouver Canada; ^28^ Département de Neurologie/UF Neuro‐Métabolisme Centre de Référence des Maladies Métaboliques et Lysosomales Neurologiques (CRML‐Neuro), Hôpital Pitié‐Salpêtrière Paris France; ^29^ INSPIRE‐PNRM+, Neuroimaging Center (NIC) University Medical Center of the Johannes Gutenberg University Mainz Mainz Germany; ^30^ Genomic Medicine Manchester University National Health Service Foundation Trust Manchester UK; ^31^ Clinical Science for LSD SphinCS Hochheim Germany; ^32^ Westmead Hospital Westmead New South Wales Australia; ^33^ Neuropsychiatry Royal Melbourne Hospital Parkville Australia; ^34^ Lysosomal and Rare Diseases Research and Treatment Center (LDRTC) Fairfax Virginia USA; ^35^ University Hospitals Birmingham NHS Foundation Trust Birmingham UK; ^36^ International Niemann‐Pick Disease Alliance Washington Tyne and Wear UK; ^37^ Niemann‐Pick UK Washington Tyne and Wear UK; ^38^ Department of Pediatrics, Neurological Sciences, and Anatomy/Cell Biology Rush University Medical Center Chicago Illinois USA; ^39^ Department of Neurology Mayo Clinic Rochester Minnesota USA; ^40^ Department of Pediatric and Adolescent Medicine Mayo Clinic Rochester Minnesota USA; ^41^ Department of Medical Genetics Mayo Clinic Rochester Minnesota USA; ^42^ IntraBio, Inc. Austin Texas USA

**Keywords:** diagnosis, guidelines, management, Niemann‐Pick disease type C, NPC, treatment

## Abstract

In 2018, the International Niemann‐Pick Disease Alliance (INPDA) and the International Niemann‐Pick Disease Registry (INPDR) developed and published comprehensive clinical management guidelines to support inclusive and standardized care pathways in Niemann‐Pick disease type C (NPC)—an ultra‐rare, autosomal recessive, neurovisceral lysosomal disorder. Since then, advances in diagnostics, care, and the approval of two novel disease‐modifying agents have underscored the need to revise these guidelines to ensure safe, consistent, and high‐quality care for those affected by NPC. In response, the INPDA and INPDR convened a multidisciplinary Guidelines Development Group (GDG) comprising individuals with NPC expertise from 14 countries across five continents, representing a broad range of specialties, as well as patients and families involved in NPC care. Informed by a comprehensive literature review and two meetings, the GDG systematically reviewed, revised, and updated the 2018 guideline statements, re‐evaluating the level of evidence, strength of recommendations, and expert agreement for each. The resulting 2025 consensus clinical management guidelines constitute a timely, up‐to‐date, and internationally applicable resource for the diagnosis, treatment, and holistic management of individuals with NPC. These guidelines serve as a critical resource for specialist centers, hospital‐based medical teams, staff involved in NPC patient care, family physicians and other primary caregivers, and, importantly, patients and their families.

## 1 Introduction

Niemann‐Pick disease type C (NPC) is an ultra‐rare, autosomal recessive, neurovisceral lysosomal disorder caused by pathological variants in the *NPC1* or *NPC2* genes [1, 2, 3]. Patterns of disease onset, classification systems for defining the clinical spectrum, and scales measuring disease severity are well documented [4, 5]. However, atypical presentations of NPC, non‐specific signs and symptoms, and the intersection of disease rarity and limited clinical awareness often lead to suboptimal or delayed care [6, 7, 8].

Challenges also remain in differential diagnosis, prolonged times to diagnosis, and in some cases, misdiagnosis [6, 8, 9, 10, 11]. Consequently, NPC patients may encounter delayed or restricted access to suitable management strategies. As a progressive disease, these challenges translate to worse clinical outcomes, adverse effects on individuals' overall wellbeing and quality of life (QoL), and reduced life expectancy [4, 12, 13, 14].

Symptomatic therapies remain the mainstay of NPC management, but they are increasingly complemented by disease‐modifying agents where available [7]. Miglustat is a well‐established, licensed disease‐modifying therapy for managing neurological manifestations in NPC, with research supporting its role in attenuating disease progression in some patients [15, 16, 17]. Levacetylleucine, arimoclomol, and combination therapeutic approaches with miglustat are also options for consideration for patients with a confirmed diagnosis of NPC [18, 19, 20, 21]. The introduction of these new therapeutic options highlights the need for the development and dissemination of updated clinical guidelines to support continued high‐quality care for NPC patients.

In 2018, the International Niemann‐Pick Disease Alliance (INPDA) and the International Niemann‐Pick Disease Registry (INPDR) developed and published a comprehensive set of disease management guidelines to overcome barriers to optimal NPC care and improve patient outcomes [22]. These guidelines have now been updated to reflect the latest advancements in the field and provide a structured approach to NPC management. They aim to support healthcare providers (HCPs) in understanding the needs of NPC patients and inform best practices—from initial clinical suspicion and diagnostic investigations to licensed treatment options, emerging therapies demonstrating promising efficacy in ongoing research, additional supportive care strategies, and disease monitoring. Overall, the guidelines strive to deliver high‐quality, evidence‐based, and personalized care for patients of all ages with NPC.

Best practice recommendations are particularly relevant for specialist centers, hospital‐based medical teams, family physicians, primary caregivers, and staff involved in caring for NPC patients. However, beyond their application in clinical settings, the statements outlined in this document may also serve as a valuable resource for patients and their families. Further, by offering definitive standard care practices for relevant specialists, they aim to foster collaboration across expert centers and promote a multidisciplinary approach to the treatment and holistic management of this complex disorder.

## 2 Methods

The methodology used to develop the original consensus clinical management guidelines for NPC has been previously described; the updated guidelines outlined in this paper build upon and follow a similar approach [22].

Experts from 14 countries across five continents convened to update the NPC clinical management guidelines, forming the new Guidelines Development Group (GDG). The GDG comprises individuals with NPC expertise from different specialties and patient representatives. This diverse representation ensures that the revisions to the clinical management guidelines support inclusive and standardized care pathways for NPC patients worldwide.

A comprehensive literature review was conducted using a three‐step approach. First, a PubMed search was run to identify English‐language papers on NPC published since 2018. The search included NPC acronyms, synonyms, and terms relevant to the clinical management guidelines. The literature search identified 524 abstracts, which were selected for further review and grouped according to their relevance to the 2018 NPC clinical guideline statements. The final step involved reviewing all abstracts to identify novel content that either supplemented or contradicted the 2018 NPC clinical guideline statements. Eighty‐eight abstracts were then extracted and summarized for presentation, discussion, and critical evaluation by GDG members.

The GDG held two meetings. During the first project initiation meeting, conducted virtually on January 10, 2025, the GDG established that the guideline updates would primarily focus on NPC management, specifically regarding NPC disease‐modifying therapies. The second GDG meeting involved both in‐person and online participation on February 3, 2025 in San Diego, USA. The GDG communicated via email throughout the guideline refinement process, ensuring regular opportunities for review and feedback on the proposed updates. Finally, GDG members re‐evaluated the level of evidence for each clinical guideline statement and the strength of each recommendation, as per the Grading of Recommendations, Assessment, Development and Evaluation (GRADE) methodology employed previously [22].

The competing interests of the GDG members have been recorded in writing and addressed.

## 3 Section 1: Definition and Epidemiology

### 3.1 Definition of NPC

**Statement #1**: NPC is a progressive and life‐limiting autosomal recessive, neurovisceral lysosomal disorder caused by pathological variants in either *NPC1* or *NPC2*. Variants in these genes are associated with abnormal endosomal‐lysosomal trafficking, resulting in the accumulation of unesterified cholesterol and other lipids in the lysosomes. Disease onset varies, ranging from antenatally to throughout the lifespan.

- *Strength of recommendation: 1*
- *Level of evidence: A*
- *Expert's opinion: completely agree (96.55%), mostly agree (3.45%), partially agree (0.00%), partially disagree (0.00%), mostly disagree (0.00%), and completely disagree (0.00%)*.

Niemann‐Pick disease type C (OMIM#257220; OMIM#607625) is a lysosomal storage disorder caused by variants in either the *NPC1* or *NPC2* genes [1, 2, 23, 24]. The two genes code for their respective proteins, NPC1 and NPC2 [25, 26, 27]. These proteins work sequentially to facilitate the bidirectional cellular trafficking of cholesterol and other lipids from the lysosome to the endoplasmic reticulum [28, 29, 30]. Variants that cause disease in either gene lead to tissue accumulation of multiple lipids (see Reference [31] for review). NPC disease is a progressive disorder characterized by neuro‐visceral manifestations that can appear at any age, from the antenatal period through adulthood. Life expectancy among patients with NPC disease varies depending on the age at neurological symptom onset, ranging from a few days to many decades [4, 14, 32, 33, 34, 35, 36].

### 3.2 How Common Is NPC Disease?

**Statement #2**: NPC disease is ultra‐rare, with an estimated incidence of one case per 100 000 live births. The disease is pan‐ethnic, with at least 95% of all cases attributed to variants in *NPC1*, and the remainder in *NPC2*. The milder forms of the disease remain underdiagnosed.

- *Strength of recommendation: 2*
- *Level of evidence: C*
- *Expert's opinion: completely agree (75.86%), mostly agree (24.14%), partially agree (0.00%), partially disagree (0.00%), mostly disagree (0.00%), and completely disagree (0.00%)*.

Retrospective, national expert center‐based studies from Australia, the Netherlands, the UK, Portugal, the Czech Republic, France, and the United Arab Emirates have reported an annual incidence varying between 0.25 and 2.20 per 100 000 live births [14, 37, 38, 39, 40, 41]. Published incidence data that includes information prior to 1990 may underestimate the disease prevalence. These figures should be compared with later studies that suggest a higher incidence due to improved diagnostics and awareness, leading to the recognition of more adult‐onset cases [12]. Additionally, data compiled from more recent parallel large exome sequencing from four independent datasets established a combined incidence of NPC of 1/89 229, or 1.12 affected patients per 100 000 conceptions [8]. Interestingly, in the Wassif et al. [8] study, the inclusion of two variants of controversial pathogenicity suggests a much higher incidence in the range of 1/40 000 of still‐unrecognized late‐onset or milder forms. Indeed, attenuated phenotypes may not be clinically suspected or may be missed by diagnostic laboratories.

## 4 Section 2: Clinical Presentation

### 4.1 How Can NPC Disease Be Best Classified?

**Statement #3**: The clinical manifestations and life expectancy of NPC patients vary markedly and reflect a continuum. In neonates and children, NPC may initially present as a systemic disease with subtle neurological manifestations. Nevertheless, NPC is best classified according to the age at onset of neurological manifestations, as this correlates with prognosis, as follows:

1. Pre/perinatal (< 2 months)
2. Early infantile (2 months–2 years)
3. Late infantile (2–6 years)
4. Juvenile (6–16 years)
5. Adult (> 16 years)
  - *Strength of recommendation: 1*
  - *Level of evidence: B*
  - *Expert's opinion: completely agree (75.86%), mostly agree (20.69%), partially agree (3.45%), partially disagree (0.00%), mostly disagree (0.00%), and completely disagree (0.00%)*.

The clinical spectrum of NPC disease ranges from a pre/perinatal, rapidly progressive, and fatal disorder to an adult‐onset, chronic neurodegenerative disease. Data from a large cohort of French NPC patients and the INPDR indicate that the age at onset of neurological symptoms predicts disease severity and life expectancy [4, 14]. Disease classifications based on the age of onset of the first neurological symptom may be used to guide clinicians in daily care, genetic counseling, and estimating disease trajectory. There is an overlap between the neurological forms, as NPC disease comprises a continuum [12]. The relative distributions of the five age categories based on the national/international registry are listed in Table 1.

**TABLE 1:** Distribution of clinical forms of NPC disease in large cohorts.

Studies	Acute perinatal form (%)	Early infantile neurological onset (%)	Late infantile neurological onset (%)	Juvenile neurological onset (%)	Adult neurological onset (%)	Total no.
France + European countries [42]	12	30	23	30	5	125
Spain [43, 44, 45]	7	37	21	25	11	57
Italy [46, 47]	7	26	32	23	12	43
France [14]	9	26	22	26	16	107
Germany [48]	3	3	35	54	5	37
Czech Republic [34]	6	17	24	37	17	54
UK [35]	5	6	39	32	19	132
European countries [12]	—	11	31	31	27	145

There are atypical NPC presentations that fall outside the classification system based on the age of neurological symptom onset, which constitute a small but significant proportion of cases. These include two key visceral forms of NPC:

1. The acute pre/perinatal form (fetal hydrops or ascites, early liver, multi‐organ failure, and in some cases, respiratory failure), which usually results in death before the age of 6 months.
2. The isolated systemic/visceral form occurs in older pediatric or adult patients presenting with isolated hepatosplenomegaly and having either no neurological symptoms or a significant latency before their onset.

The global contribution of these forms has rarely been calculated, and such patients are typically not enrolled in registries. Two features emerge from the compiled data presented in Table 1: the early infantile neurological onset form appears more frequent in Southern Europe and the Middle East, and patients with the adult‐onset neurological form seem to represent at least 20% of the cases of NPC and, owing to their longer survival, probably constitute the largest patient group in terms of disease prevalence [12, 14].

## 5 Is the Clinical Presentation Different in Specific Age Groups?

### 5.1 Pre/Perinatal (< 2 Months)

**Statement #4**: NPC primarily manifests in the pre/perinatal age group as liver disease presenting with prolonged cholestatic jaundice, hepatosplenomegaly, and in some cases fetal hydrops or ascites and acute liver failure, with or without pulmonary disease.

- *Strength of recommendation: 1*
- *Level of evidence: B*
- *Expert's opinion: completely agree (96.55%), mostly agree (3.45%), partially agree (0.00%), partially disagree (0.00%), mostly disagree (0.00%), and completely disagree (0.00%)*.

NPC disease presentation during the pre/perinatal period varies, with the most common presentation being prolonged cholestatic jaundice and mild hepatosplenomegaly [6]. In most cases, jaundice resolves spontaneously by 3–4 months of age, while organomegaly persists to variable degrees. In approximately 8%–9% of cases, hepatic manifestations may progress rapidly to acute liver and/or multi‐organ failure, subsequently leading to death within 6 months [32]. Rarely, the initial presentation may be fetal hydrops or ascites. The rapidly progressing cohort may have associated neurological presentations such as failure to thrive and hypotonia [33].

### 5.2 Early Infantile (2 Months to 2 Years)

**Statement #5**: Hypotonia and delay in developmental motor milestones characterize the neurological manifestation of NPC in early infancy. Hepatosplenomegaly and/or prolonged neonatal jaundice are almost invariably noted, although cases with severe hypotonia in early infancy without obvious visceral disease can occur. Pulmonary disease may also be present.

- *Strength of recommendation: 1*
- *Level of evidence: B*
- *Expert's opinion: completely agree (86.21%), mostly agree (10.34%), partially agree (3.45%), partially disagree (0.00%), mostly disagree (0.00%), and completely disagree (0.00%)*.

### 5.3 Late Infantile (2–6 Years)

**Statement #6**: Clumsiness, gait disturbance, and fine motor skill impairments characterize this age of disease onset. Speech delay, a history of neonatal cholestasis, seizures, pulmonary disease, variable visceromegaly, and failure to reach or loss of developmental milestones may be noted. Vertical supranuclear saccadic palsy (VSSP), followed later by vertical supranuclear gaze palsy (VSGP), is typically present but often unrecognized. The first symptoms may be gelastic cataplexy (sometimes associated with narcolepsy) or sensory deafness.

- *Strength of recommendation: 1*
- *Level of evidence: B*
- *Expert's opinion: completely agree (86.36%), mostly agree (13.64%), partially agree (0.00%), partially disagree (0.00%), mostly disagree (0.00%), and completely disagree (0.00%)*.

### 5.4 Juvenile (6–16 Years)

**Statement #7**: Juvenile onset manifests as cognitive impairment (lagging behind peers in school, language and learning difficulties), coordination problems (clumsiness, frequent falls, progressive ataxia, and dystonia), seizures, and VSGP.

- *Strength of recommendation: 1*
- *Level of evidence: B*
- *Expert's opinion: completely agree (86.36%), mostly agree (13.64%), partially agree (0.00%), partially disagree (0.00%), mostly disagree (0.00%), and completely disagree (0.00%)*.

### 5.5 Adult (> 16 Years)

**Statement #8**: Adult‐onset NPC patients may represent up to a third of all NPC patients. Cognitive impairment invariably occurs and tends to present with higher rates of psychiatric illness alongside neurological manifestations. Diagnostic delay is common but minimized if the characteristic VSGP is identified.

- *Strength of recommendation: 1*
- *Level of evidence: B*
- *Expert's opinion: completely agree (65.52%), mostly agree (31.03%), partially agree (3.45%), partially disagree (0.00%), mostly disagree (0.00%), and completely disagree (0.00%)*.

The age of onset of NPC varies significantly across the lifespan (Table 2) [14]. However, patients are increasingly recognized as presenting with late‐onset illness in adolescence and early and mid‐adulthood and may present as late as the seventh decade [49]. Early development is often normal, with children achieving all developmental milestones appropriate for their age. Despite this, a high proportion of adult‐onset NPC patients have intellectual disabilities or learning disorders [50]. In the two largest international registries, the adult‐onset form occurred in 27% of all NPC patients [4, 12].

Patients in this age group are less likely to present with seizures, gelastic cataplexy, and diagnosed visceral disease. Some patients may have had previous symptoms that began several years before the onset of the chronic neurodegenerative disease, such as hepatomegaly or splenomegaly, learning disorders in childhood, childhood dementia, and hearing and speech defects. In adult‐onset patients, diagnostic delay of five or more years is common, although this delay may be minimized when the more specific symptom of VSGP is recognized [51].

**TABLE 2:** Summary of clinical signs and symptoms in NPC, by age of onset.

Age at onset	Systemic manifestations	Neurological/psychiatric manifestations
Pre/perinatal (< 2 months)	Fetal ascites/hydrops Hepatosplenomegaly Cholestatic jaundice Thrombocytopenia Pulmonary disease Liver failure Failure to thrive	Hypotonia
Early infantile (2 months to 2 years)	Hepatosplenomegaly or splenomegaly (isolated, or with neurological manifestations) Prolonged neonatal jaundice Pulmonary disease	Central hypotonia Delayed developmental motor milestones, speech delay Dysphagia, spasticity VSGP
Late infantile (2 to 6 years)	Hepatosplenomegaly or splenomegaly (isolated, or with neurological manifestations) History of prolonged neonatal cholestatic jaundice Pulmonary disease	Developmental delay/regression, speech delay Clumsiness, frequent falls Progressive ataxia, dystonia, dysarthria, dysphagia Seizures Gelastic cataplexy VSGP Hearing loss VSSP
Juvenile (6 to 16 years)	Hepatosplenomegaly or splenomegaly (isolated, or with neurological manifestations; often not present) Liver disease, including progression to cirrhosis	Poor school performance, learning disability Loss of language skills Frequent falls, clumsiness Progressive ataxia, dysarthria, dystonia, dysmetria, dyskinesia, dysphagia VSGP Gelastic cataplexy Seizures Behavioral problems
Adult (> 16 years)	Splenomegaly (often not present; isolated in very rare cases) Inflammatory bowel disease Interstitial lung disease Liver disease, including progression to cirrhosis	Early‐onset cognitive decline, dementia, learning disability Atypical psychiatric signs: schizophrenia (psychosis), depression (psychiatric symptoms often predate neurological manifestations, and may be treatment resistant) Clumsiness, progressive motor symptoms, tremor, ataxia, dystonia/dyskinesia Dysarthria and dysphagia (may occur later) VSGP

### 5.6 Differential Diagnosis of NPC

**Statement #9**: The signs and symptoms of NPC are nonspecific, which may lead to misdiagnosis. However, the presence of cataplexy and/or saccadic eye movement abnormalities should raise suspicion of NPC and help differentiate NPC from other neurodegenerative disorders.

- *Strength of recommendation: 1*
- *Level of evidence: B*
- *Expert's opinion: completely agree (68.97%), mostly agree (27.59%), partially agree (3.45%), partially disagree (0.00%), mostly disagree (0.00%), and completely disagree (0.00%)*.

As clinical indicators of NPC vary with age at disease onset and are not disease‐specific, a process of differential diagnosis is necessary to distinguish NPC from other conditions. Table 3 presents examples of potential conditions that exhibit similar symptoms to NPC within each age group, but it is not exhaustive.

**TABLE 3:** Conditions raising the suspicion of NPC and differential diagnosis.

Age group	Clinical indicators [6, 7]	Differential diagnosis [6, 9, 10, 11, 52]
Early infancy (< 2 years)	Prolonged cholestatic neonatal jaundice Hepatosplenomegaly Pulmonary disease Developmental delay Isolated splenomegaly (± hepatomegaly)	Bile acid synthesis disorders (BASD) Peroxisomal disorders Idiopathic neonatal hepatitis Wolman disease Acid sphingomyelinase deficiency (ASMD) Gaucher disease type II/III Cerebrotendinous xanthomatosis (± developmental delay) Congenital defects of glycosylation
Late infantile and juvenile (2–16 years)	Clumsiness Poor school performance Progressive ataxia Dysarthria Dystonia Gelastic cataplexy VSGP Isolated splenomegaly (± hepatomegaly)	Age‐appropriate neurodegenerative disorders, including: Wilson disease GM1 or GM2 gangliosidoses Neuronal ceroid‐lipofuscinosis Amino acidurias & organic acidopathies Hereditary disorders with periodic paralysis Early‐onset dementia Primary psychiatric disorders Krabbe disease Metachromatic leukodystrophy X‐linked adrenoleukodystrophy
Adults (> 16 years)	Atypical psychotic disorder or progressive neurological syndrome, including: Ataxia Dystonia Cognitive decline Dysarthria VSGP (± splenomegaly) Isolated splenomegaly (± hepatomegaly)	Late‐onset neurodegenerative disorders^a^ such as: Huntington's disease Frontotemporal dementia Wilson disease Cerebrotendinous xanthomatosis GM1 or GM2 gangliosidoses Friedreich Ataxia Progressive supranuclear palsy (rare, late‐adult‐onset)

^a^ Notably, contrary to several of the neurodegenerative disorders listed, NPC patients do not exhibit peripheral neuropathy, and brain structure—as shown by magnetic resonance imaging (MRI)—is normal or shows nonspecific abnormalities (mainly atrophy).

The Niemann‐Pick disease type C Suspicion Index (NPC‐SI) has been developed as a simple, interactive screening tool to assist HCPs in the early identification and referral of individuals suspected of NPC [53]. The NPC‐SI includes two age‐specific screening tools: one for individuals older than 4 years and another for individuals younger than four (early‐onset NPC). Both tools use a point system, whereby key signs and symptoms are scored and ranked based on their strength of association with NPC, allowing an overall risk prediction score to be calculated.

### 5.7 NPC Disease Severity Scales

**Statement #10**: NPC‐specific disease severity scales are useful adjuncts to clinical judgment in assessing disease burden, trajectory, and response to therapy.

- *Strength of recommendation: 1*
- *Level of evidence: B*
- *Expert's opinion: completely agree (62.07%), mostly agree (34.48%), partially agree (3.45%), partially disagree (0.00%), mostly disagree (0.00%), and completely disagree (0.00%)*.

Clinical assessment of disease severity relies on the treating clinician's experience with a specific condition. Consequently, assessing rare diseases is inherently more challenging due to limited clinician exposure.

NPC‐specific scales assess disease severity, trajectory, and response to therapy based on neurological impairments, generating a composite score. In the previous guidelines, three severity scoring systems were identified: the NPC clinical database (NPC‐cdb), the Niemann‐Pick disease type C Clinical Severity Scale (NPC‐CSS), and the modified disability scale [48, 54]. The annual severity increment score (ASIS) has recently emerged as another scale that may be useful in assessing disease trajectory in the clinical trial setting [55].

Over the last decade, a version of the NPC‐CSS has been used as a primary or secondary endpoint in nearly all NPC clinical trials. Multiple studies support the use of a shorter form of the original, comprehensive 17‐point NPC‐CSS, identifying speech, swallowing, fine motor skills, ambulation, and cognition as the most clinically relevant measures for clinicians and patients [5, 56, 57]. Consequently, a shorter 4‐ or 5‐domain NPC‐CSS is consistently used in clinical trials, and a fully validated version of the 5‐domain scale, generated with input from NPC clinicians and the U.S. Food and Drug Administration (FDA), is a priority for the community and is currently undergoing formal validation studies.

In addition to the NPC‐CSS, the SARA test (Scale for Assessment and Rating of Ataxia) has also been used in recent NPC clinical trials, although this measure only addresses ataxia/motor impairments and is not validated in children under 2 years of age [58, 59, 60, 61].

In a clinical setting, assessing disease severity every 6–12 months using an NPC disease‐specific scale provides valuable information for clinicians, patients, and the community if captured in a registry. While any of the NPC scales can be used, clinicians may find that the NPC‐CSS provides a more direct comparison to clinical trial data [5].

### 5.8 Is Saccadic Eye Movement Evaluation a Measure of Disease Status?

**Statement #11**: Measures of horizontal saccadic peak velocity and latency, vertical saccadic duration and amplitude, and horizontal position smooth pursuit are robust objective measures of disease status with little inter‐rater variability.

- *Strength of recommendation: 2*
- *Level of evidence: B*
- *Expert's opinion: completely agree (45.45%), mostly agree (18.18%), partially agree (36.36%), partially disagree (0.00%), mostly disagree (0.00%), and completely disagree (0.00%)*.

Previously, VSGP (impaired ability to generate saccades and smooth pursuit), but not VSSP (impaired ability to generate vertical saccades), was noted as the hallmark symptom of NPC at all disease stages. These are usually seen early in disease progression, apart from the early neonatal form, in which they are often seen later in the neurological disease progression. However, quantitative measurements of eye movements have established VSSP as the cardinal sign of NPC, and it usually progresses to VSGP [62]. Measures of horizontal saccadic peak velocity and latency, vertical saccadic duration and amplitude, and horizontal position smooth pursuit provide robust, objective indicators of disease status in NPC and correlate with structural brain changes [62]. Among these, horizontal saccadic gain and self‐paced saccades may be particularly sensitive markers in adults. Horizontal saccadic gain is strongly associated with pontine area and parietal eye field volume on MRI, while self‐paced saccades reflect frontal eye field integrity, suggesting their potential as reliable biomarkers of disease progression [63, 64].

## 6 Section 3: Investigations

**FIGURE 1:**
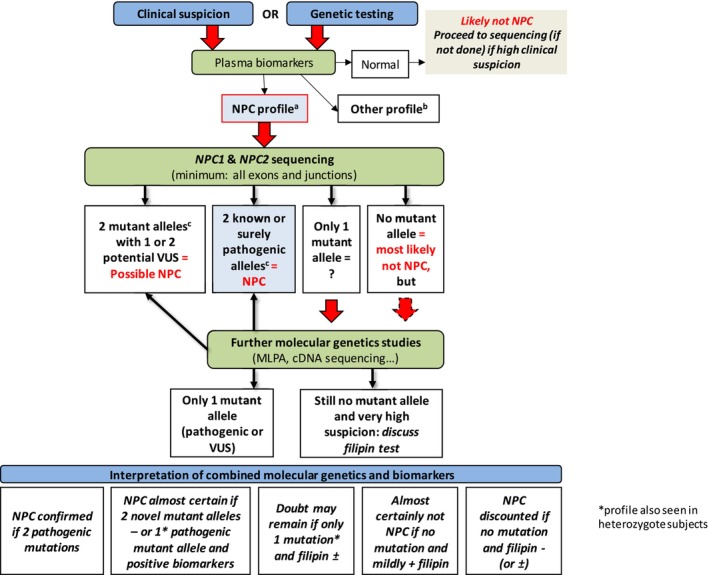
Niemann‐Pick disease type C laboratory diagnosis algorithm. What investigations should be performed in an individual suspected of NPC? Modified from: Patterson et al. [67, 71]. CDNA, Complementary DNA; MLPA, Multiplex Ligation‐dependent Probe Amplification (evaluates copy number changes, allows detection of large deletions or false homozygous status with a deletion on the other allele); VUS, Variant of unknown significance; WES, Whole exome sequencing; WGS, Whole genome sequencing. ^a^Elevated cholestane‐triol or bile acid derivative and/or PPCS, with normal or slightly elevated lyso‐SM. ^b^Cholestane‐triol is also elevated in ASMD, acid lipase deficiency, cerebrotendinous xanthomatosis, and certain neonatal cholestasis conditions. All lyso‐SM analogues and bile acid derivatives are elevated in ASMD. ^c^Check allele segregation by parental study or other test.

Once NPC is suspected, diagnosis can be confirmed through a combination of biochemical and molecular genetic studies, depending on the availability of various diagnostic tools [65, 66]. In recent years, several plasma metabolites have been identified as sensitive and specific diagnostic biomarkers for NPC. These include cholestane‐3β, 5α, 6β‐triol, the N‐palmitoyl‐O‐phosphocholine‐serine (PPCS):lyso‐sphingomyelin (lyso‐SM) ratio, and bile acid metabolites, specifically 3β‐sulfooxy‐7β‐N‐acetylglucosaminyl‐5‐cholen‐24‐oic acid and its glycine and taurine conjugates in urine [57, 65, 67, 68, 69, 70]. Figure 1 describes a laboratory diagnostic algorithm for NPC.

### 6.1 Biomarkers

**Statement #12**: Biomarker assessment should be considered the first‐line test to screen for NPC. Three classes of biochemical markers are currently in use (oxysterols, PPCS, and lyso‐SM) or development (bile acid derivatives). They can be used alone or together to enhance sensitivity and specificity. Typically, the diagnosis must be confirmed by deoxyribonucleic acid (DNA) sequencing, although diagnostic routes depend on tool availability.

- *Strength of recommendation: 1*
- *Level of evidence: B*
- *Expert's opinion: completely agree (55.17%), mostly agree (27.59%), partially agree (17.24%), partially disagree (0.00%), mostly disagree (0.00%), and completely disagree (0.00%)*.

#### 6.1.1 Oxysterols (Cholesterol Oxidation Products)

The oxysterols cholestane‐3β, 5α, 6β‐triol (C‐triol) and 7‐ketocholesterol (7‐KC) are sensitive markers of NPC, though C‐triol is more specific [72, 73, 74, 75].

#### 6.1.2 Lyso‐Sphingolipids and PPCS

The simultaneous mass spectrometric measurement in plasma of lyso‐SM (also known as sphingosyl‐phosphorylcholine) and lyso‐SM 509, known as PPCS, appears to be a useful diagnostic tool [70, 76, 77, 78, 79, 80, 81]. Striking elevations of PPCS have been reported in both NPC and ASMD, with high sensitivity for detecting both disorders but poor distinction between them. For lyso‐SM, a large increase only occurs in ASMD, with marginal or no elevation in NPC. Therefore, the combined assay of PPCS and lyso‐SM provides good discrimination between NPC and ASMD [82, 83]. A high PPCS/lyso‐SM ratio appears to be very specific to NPC.

#### 6.1.3 Bile Acids

Several unusual bile acid species in plasma and urine have been identified in NPC. The most important analytical species is 3β, 5α, 6β‐trihydroxy‐cholanoyl‐glycine, detectable in plasma and dried blood spots [84, 85, 86]. It is highly sensitive and more specific than oxysterols (only elevated in ASMD and NPC).

### 6.2 Molecular Genetic Studies

**Statement #13**: Any individual whose diagnosis of NPC is considered based on their clinical manifestation and/or abnormal biomarker profile should undergo genetic testing for NPC genes to confirm the diagnosis. Referral to a clinical geneticist or genetic counselor should be considered following NPC diagnosis.

- *Strength of recommendation: 1*
- *Level of evidence: A*
- *Expert's opinion: completely agree (93.10%), mostly agree (3.45%), partially agree (3.45%), partially disagree (0.00%), mostly disagree (0.00%), and completely disagree (0.00%)*.

In some national jurisdictions, gene panels that include *NPC1* and *NPC2* gene testing have increasingly become the first‐line test for assessing patients with signs and symptoms of neurodegeneration.

Genetic testing (*NPC1* and *NPC2*) is recommended to confirm NPC diagnosis [11, 87, 88]. Further, it is the only reliable method to diagnose NPC carriers within the family and the highly preferred strategy for prenatal diagnosis. Furthermore, it may help establish genotype–phenotype correlations. However, some genetic changes (e.g., deep intronic variants, complex structural variants) may not be identified by routine sequencing methods and require complementary testing [71]. In some cases, genetic reanalysis may also be needed to provide a more accurate diagnosis. Nonetheless, a small proportion of *NPC1* alleles remain unidentified in patients diagnosed with NPC through other clinical tests. If possible, segregation of the alleles should be confirmed in the parents. The identification of two alleles with known disease‐causing variants in either the *NPC1* or *NPC2* gene confirms the diagnosis of NPC. To date, about 700 different *NPC1* variants have been reported, among which around 420 are considered pathogenic, with only a limited number of common or recurrent (often in certain populations) variants [89].

### 6.3 Neuroimaging

Neuroimaging in NPC is an emerging area of research that provides valuable insights into disease progression, though its full diagnostic potential is not yet fully understood.

Imaging data gathered primarily from adults with NPC reveals a variable pattern. Some patients demonstrate normal imaging findings early in the disease, while most develop cerebellar atrophy, which correlates with ataxia and ocular‐motor dysfunction [90]. Volume loss in the hippocampus, basal ganglia, and thalamus also relates to disease progression [91].

White matter involvement is described in infantile forms of NPC, often widespread and appearing as diffusion imaging changes [92] or corpus callosum atrophy [93]. An increased pontine‐to‐midbrain ratio, similar to that seen in progressive supranuclear palsy but to a lesser degree, has also been observed [94].

In some patients, atrophy predominantly affects the frontal and temporal lobes [95]. However, these changes are often subtle and nonspecific and may be more valuable as a biomarker than a diagnostic tool.

## 7 Section 4: Management

NPC disease is not yet curable, and optimal disease management employs a multidisciplinary team based in a specialist center, closely liaising with community care providers (Table 4). The mainstay of therapy is symptom management, employing disease‐modifying agent(s) when available.

**TABLE 4:** Multidisciplinary assessments of patients with NPC.

Discipline	Features of NPC for which this discipline may be of assistance	References
Primary care physician	Assist with general medical care; coordinate specialists; provide support for family	Expert opinion
Metabolic diseases specialist	Diagnosis of NPC and exclusion of other disorders in the differential diagnosis; ongoing patient assessment for disease progression and response to therapy	[71]
Neurologist	Gelastic cataplexy, movement disorders, dystonia, and seizures	[15]
Psychiatrist	Psychosis, behavioral disturbances, depression	[51]
Neuro‐ophthalmologist	Diagnosis (vertical gaze palsy) and assess response to therapy (changes in saccadic eye movement velocity)	[96]
Anesthesiologist	Assess for anesthetic risk as needed	[97, 98]
Neuropsychologist	Assess for cognitive involvement at baseline and in response to therapy	[99]
Speech and language therapist	Assess for dysphagia and aspiration risk; speech therapy for children	Expert opinion
Occupational and physical therapists/rehabilitation physician	Assess development and develop aids and home adjustments as needed for patients with communication and physical challenges	[100]
Orthopedic surgeon	Assess the need for surgical correction of severe scoliosis, osteo‐articular retractions, spasticity treatments and hip problems	Expert opinion
Nutritionist/gastroenterologists	Assess nutritional status in patients who may be losing weight due to dysphagia or side effects of therapy. Gastrostomy tube insertion when swallowing is unsafe Assess for inflammatory bowel disease	[101]
Social worker	Support of patients and families living with disabilities who require enhanced resources in the community	Expert opinion
Genetic counselor	Provide counseling for families as to recurrence risk and options for prenatal diagnosis if desired	[71]

**Statement #14**: Patients with NPC exhibit multisystem disease manifestations and benefit from holistic, multidisciplinary follow‐up from physicians and allied HCPs with experience in this condition. Wherever possible, patients identified with NPC should be referred to a center with expertise in the care of this condition.

- *Strength of recommendation: 1*
- *Level of evidence: A*
- *Expert's opinion: completely agree (93.10%), mostly agree (6.90%), partially agree (0.00%), partially disagree (0.00%), mostly disagree (0.00%), and completely disagree (0.00%)*.

### 7.1 Symptom Assessment and Management

A baseline assessment at diagnosis is required to determine the current level of disease severity and retrospectively estimate the rate of progression [32, 102, 103]. An interval history and NPC clinical severity score assessment are performed regularly to establish the rate of progression or response to therapy [102, 103].

For optimal symptom control and functional capacity, functional assessments should be performed at the time of diagnosis or symptom onset and at regular intervals thereafter. For more details on symptom assessments and management, see Table 5.

**TABLE 5:** Recommended NPC assessment and management.

Signs, symptoms and clinical manifestations	Recommended assessment (including frequency)	Management	References
Growth and developmental delay	The growth of children with NPC (height, weight, and head circumference) should be assessed at regular intervals (at diagnosis, then every 6–12 months) as part of routine health assessments by their primary HCP.	Adequate nutritional control and supplements should be provided if needed.	[22, 104]
		Supportive therapies should be initiated if developmental delay is observed.	
	Developmental progress should be monitored using age‐appropriate instruments.		
Mobility	Mobility, balance, core stability, trunk control, spasticity, foot posture, and strength should be assessed regularly by a suitably qualified physical therapist using well‐established scales such as SARA for ataxia.	Strategies to maintain optimal mobility and reduce falls, such as providing walking/mobility aids, ankle‐foot orthotics, and exercise programs, should be sought proactively to prolong mobility and transfer ability.	[22, 61, 105, 106]
	The Abnormal Involuntary Movement Scale (AIMS) should be used for infants under 18 months of age to identify delays or changes in motor skill development. The Bruininks‐Oseretsky Test of Motor Proficiency Second Edition (BOT‐2) may be used for follow‐up in children.		
Swallowing and diet	All patients should undergo a comprehensive clinical swallowing assessment by a speech and language therapist. A videofluoroscopic swallowing (VFS) assessment should be conducted in all patients with neurological symptoms. A pulmonary function assessment is recommended in all patients.	Dietary modification and compensatory postures may be beneficial for individuals with dysphagia. Counseling individuals on the long‐term utility of assisted feeding by gastrostomy tube is recommended early in the disease to prevent delay and improve quality of life.	[22]
	The presence of dysphagia, aspiration and response to therapy should be documented at diagnosis and then every 6 months in children and every 12 months in adults if the patient is asymptomatic with stable disease.		
	NPC patients should also undergo a nutritional review by a dietitian.		
Speech	Comprehensive communication evaluation by a speech and language therapist.	Appropriate speech and language therapy.	[22]
Spasticity	Individuals with NPC may benefit from assessments for spasticity and incipient or established contracture.	Spasticity and spasms should be treated initially by non‐pharmacological means. If these are unsuccessful, pharmacological agents, including baclofen, tizanidine, benzodiazepines, dantrolene sodium, and botulinum toxin injections, may be considered.	[22]
Bowel dysfunction and incontinence	NPC patients with bowel dysfunction should undergo screening for inflammatory bowel disease (e.g., Crohn's disease and ulcerative colitis). NPC has the highest early onset penetrance of Crohn's among monogenic diseases due to patients' predisposition to intestinal inflammation.	Consider modifying diet and lifestyle to optimize stool consistency and avoid fecal impaction and incontinence. If required, consider appropriate laxatives to optimize gut transit and stool consistency. Patients with Crohn's disease should undergo appropriate management.	[22, 107, 108]
Bladder dysfunction and incontinence	Individuals with NPC should have their history reviewed for symptoms suggestive of neurogenic bladder (recurrent urinary tract infection, nocturia, incomplete evacuation, and dribbling) and be referred for urologic evaluation if symptoms are present.	Specific treatment when necessary.	[22]
Liver dysfunction	Individuals with NPC should receive an annual general assessment of liver function. An ultrasound to monitor the size of the liver and spleen should be considered.	If cirrhosis is present, further evaluation of liver elasticity and portal flow is warranted.	[109, 110]
	Although rare in childhood, patients with NPC might have an increased risk of hepatocellular carcinoma (HCC). NPC patients with liver dysfunction may require an alpha‐fetoprotein (AFP) test and ultrasound to diagnose and stage HCC.		
Cataplexy	Cataplexy, usually without narcolepsy, is a common and specific manifestation of NPC. Early recognition of this condition is important and may support timely NPC diagnosis.	Cataplexy should be managed promptly as per local/national management guidelines. Protriptyline, other tricyclic agents, methylphenidate or modafinil may be efficacious for cataplexy. In some cases, cataplexy may be drug‐resistant.	[22, 111]
Seizures	Patients with NPC commonly experience seizures.	Seizures should be treated by a neurologist aware of the disease, considering the possibility of aggravation with some anti‐seizure drugs like carbamazepine and vigabatrin. Seizures are often difficult to treat and may require multiple drugs to control.	[22, 112]
Cognitive decline	Individuals with NPC benefit from regular evaluation of their cognitive function (at diagnosis and every 12 months), and consideration should be given to changes in their cognitive ability that may impact independence/school/work and daily living activities. Testing should be age and functionally appropriate, using standardized assessment tools.	Strategies to ensure the safety of the patient's environment and the availability of support mechanisms are essential to improve the quality of life of the patient.	[22]
Mental wellbeing	There is an increased prevalence of behavioral problems and other psychiatric disorders, such as anxiety, depression, or psychosis, in NPC. There should be a low threshold for referral to a clinical psychology/psychiatric team as appropriate at diagnosis, with check‐ups every 6 to 12 months.	Both non‐pharmacological and/or pharmacological treatments should be considered for NPC patients with mental health conditions.	[22]
Hypersalivation/drooling	Individuals with NPC are at increased risk of hypersalivation/drooling.	Patients should be treated with established interventions, including postural drainage ±pharmacological agents such as hyoscine hydrobromide transdermal patches; glycopyrronium bromide orally, subcutaneously or via a gastrostomy and small doses of orally administered atropine, or parotid/submandibular glandular injections of botulinum toxin.	[22]
Hearing	Hearing assessments should be performed at the time of diagnosis and every 12 months to document the presence of hearing loss.	When appropriate, patients should be offered hearing devices to improve general communication.	[22]

### 7.2 Disease‐Modifying Therapy

For the purpose of this paper, disease‐modifying therapy in NPC is defined as an intervention that favorably alters the rate of disease progression, improving quality of life and ultimately extending lifespan beyond that predicted by available natural history data. To understand the effects of disease‐modifying therapy in NPC, information about the natural history of disease progression is required.

#### 7.2.1 NPC Natural History

Natural history studies of NPC patient cohorts have revealed how the disease progresses over time without intervention. As summarized in Table 6, these observational data reflect the wide phenotypic variability reported in the literature [4, 32, 35, 102].

**TABLE 6:** Natural history studies of NPC.

Study title	Data collected and study cohort	Key findings
The natural history of Niemann‐Pick disease type C in the UK [32]	Clinical signs and symptoms at presentation and subsequent clinical course of all known NPC patients in the UK between 1999 and 2006 (from a patient database maintained by the Niemann–Pick Disease Group (UK) Clinical Nurse Specialist)	58 patients were still alive at the time of the paper.
		Age at diagnosis ranged from the prenatal period (with hydrops fetalis) up to 51 years.
		The paper confirmed the phenotypic variability reported elsewhere.
		Most patients in this series who survived childhood inevitably suffered neurological and, in some cases, neuropsychiatric deterioration.
	Full known UK NPC cohort (94 patients).	
Natural history of Niemann‐Pick disease type C in a multicenter observational retrospective cohort study [102]	The rate of neurological disease progression was investigated using a composite NPC scoring system, where the maximum score of four indicates severe disease.	85.7% of patients who were followed for more than 1 year showed neurological disease progression.
		23 children under 6 years of age were included, four of whom had a normal evaluation, suggesting they might have had a late‐onset phenotype.
	57 NPC patients from six countries (the majority from the UK and France).	
		The rate of neurological progression was 0.12 points per year (confidence interval (CI) 0.09–0.15).
		The rates of progression correlated with age at diagnosis, with younger patients showing the greatest progression of disease.
Observational cohort study of the natural history of Niemann‐Pick disease type C in the UK: a 5‐year update from the UK clinical database [35]	Data on patients' clinical signs and symptoms, medical history and genetic studies, summarized using descriptive methods.	Wide phenotypic variability reported.
		72 patients (49%) were still alive at the end of the observation period.
		114 (98%) had *NPC1* mutations, and 2 (2%) had *NPC2* mutations.
	Full known UK NPC cohort (146 patients).	
		5% early‐infantile, 35% late‐infantile, 29% juvenile, 17% adolescent/adult.
		14 patients diagnosed based on visceral symptoms and/or sibling history, confirmed in most cases by genetic analysis, did not have any neurological manifestations at last follow up (11 patients with mean [SD] age at last follow up 2.5 [1.8] years: 3 with mean [SD] age at death 20.8 [15.9] years).
		35% received miglustat therapy; mean treatment duration: 2.6 years (SD 2.3).
Clinical disease characteristics of patients with Niemann‐Pick Disease Type C: findings from the International Niemann‐Pick Disease Registry (INPDR) [4]	Demographic, genetic and clinical data from NPC patients enrolled in the INPDR from September 2014 to December 2019. 203 NPC patients from six European countries.	Mean age at diagnosis: 11.2 years (SD 14.2).
		168 patients had neurological symptoms: 24.2% early‐infantile, 26.4% late‐infantile, 23.0% juvenile, 20.8% adult‐onset; 5.6% had neonatal rapidly fatal systemic form.
		Most common *NPC1* variant: c. 3182T>C (35.1% of patients with known variants).
		Hepatomegaly and neonatal jaundice were most frequent in early/late‐infantile forms.
		Splenomegaly was common across all patients, present in 80% of adult‐onset cases.
		Top neurological symptoms: cognitive impairment (78.5%), dysarthria and ataxia (75.9%), VSGP (70.9%), dysphagia (69.6%).
		Moderate–severe disability was seen across all six domains of a composite disability scale, except for swallowing and seizures.
		Later neurological onset was linked to later diagnosis and death.
		Miglustat was used in 62.4% of patients.
		Common symptomatic therapies included: antiepileptics (32.9%), antidepressants (11.8%), antacids (9.4%).

#### 7.2.2 Miglustat

Miglustat, a substrate reduction therapy, is licensed as a disease‐modifying medicine to treat the neurological manifestations of patients with NPC disease. In some patients, miglustat has been shown to attenuate disease progression and improve survival.

##### 7.2.2.1 Miglustat Start Criteria

**Statement #15**: All patients with a confirmed diagnosis of NPC should be considered for miglustat therapy.

- *Strength of recommendation: 1*
- *Level of evidence: B*
- *Expert's opinion: completely agree (44.83%), mostly agree (44.83%), partially agree (10.34%), partially disagree (0.00%), mostly disagree (0.00%), and completely disagree (0.00%)*.

##### 7.2.2.2 Pivotal Clinical Trial Data

In a phase I/II trial, 29 NPC patients aged ≥ 12 years were randomized 2:1 to receive miglustat 200 mg three times daily or standard care for 1 year, with adult patients offered a 1‐year extension on active treatment [96]. The primary endpoint—change in horizontal saccadic eye movements (HSEM)—was assessed at baseline and 12 months. Swallowing was assessed at screening, 6, and 12 months, and neurological and QoL assessments were evaluated at screening and every 3 months thereafter.

At 12 months, HSEM velocity improved in patients receiving miglustat versus standard care; results were significant when excluding those taking benzodiazepines (*p* = 0.028). Children showed similar improvements in HSEM velocity at 12 months. In patients ≥ 12 years, additional benefits included improved swallowing, stable auditory function, and slower ambulatory decline.

The most common adverse events (AEs) were diarrhea (85%), flatulence (70%), and weight loss (65%). Treatment was discontinued in one pediatric patient (memory impairment) and two adults (confusion; diarrhea). No deaths were reported. The study concluded that miglustat was safe and improved or stabilized several clinically relevant NPC markers [96].

##### 7.2.2.3 Long‐Term Data

An observational study of 789 patients with neurological‐onset NPC across five national cohorts showed that miglustat treatment was associated with significantly reduced mortality risk across all ages. Median survival was extended by ~10 years from neurological onset and ~5 years from diagnosis compared to untreated patients [113].

In a cohort of 50 NPC patients, miglustat was associated with stabilized swallowing function and reduced aspiration risk [114].

##### 7.2.2.4 Differences in Treatment Efficacy by NPC Classification

Evidence for miglustat efficacy in pre/perinatal and early infantile cases remains limited [115]. In a French study of 20 children, 75% with late infantile onset (neurologic symptom onset < 6 years) showed stabilized or improved NPC disability scores, but no early infantile cases (< 2 years) demonstrated a positive neurological outcome [100]. Of nine children treated before age four, only one showed stabilization. Further data are needed to assess efficacy in this age group.

A retrospective study of early infantile neurological NPC patients in France between 1990 and 2013 included 10 treated and 16 untreated patients [115]. The median survival age was 4.42 years in the untreated group and 5.56 years in the treated group; the Kaplan–Meier survival curves were not significantly different (*p* = 0.11). The 22 remaining patients had died by study end, and none survived beyond the age of 7.4 years.

#### 7.2.3 Levacetylleucine

Levacetylleucine (also known as L‐acetylleucine) is a modified amino acid derivative of leucine, approved by the FDA for treating neurological manifestations of NPC in both adult and pediatric patients.

##### 7.2.3.1 Levacetylleucine Start Criteria

**Statement #16**: All patients with a confirmed diagnosis of NPC and neurological symptoms should be considered for levacetylleucine therapy.

- *Strength of recommendation: 2*
- *Level of evidence: B*
- *Expert's opinion: completely agree (41.38%), mostly agree (37.93%), partially agree (17.24%), partially disagree (3.45%), mostly disagree (0.00%), and completely disagree (0.00%)*.

##### 7.2.3.2 Pivotal Clinical Trial Data

In a multinational phase IIb trial, levacetylleucine improved symptoms, functioning, and QoL in children and adults with NPC after 6 weeks [13]. A subsequent phase 3, randomized, placebo‐controlled trial confirmed improved neurologic status over 12 weeks: mean SARA score change from baseline was −1.97 ± 2.43 with levacetylleucine versus −0.60 ± 2.39 with placebo (least‐squares mean difference: −1.28; 95% CI −1.91 to −0.65; *p* < 0.001) [18]. Eighty‐five percent of the patients in this study had previously received miglustat and continued its use throughout the trial. Outcomes were comparable between those receiving levacetylleucine with or without miglustat, though the latter showed slightly better improvement compared to patients who did not receive miglustat (mean change in SARA score: −2.06 vs. −1.95 with combination therapy) [116]. AE rates were similar between levacetylleucine and placebo, with no treatment‐related serious AEs reported [18].

##### 7.2.3.3 Extension Study

Long‐term data from a subsequent open‐label extension (OLE) support levacetylleucine's sustained efficacy and safety, indicating a disease‐modifying effect [117]. At 18 months, the mean (SD) change from baseline in the 5‐domain NPC‐CSS was −0.067 (2.94) with levacetylleucine, compared to 2.25 (4.74) in natural history controls (*n* = 31; aged 2–18 years) reported by Mengel et al. [117] (95% CI −4.17 to −0.46; *p* = 0.017), indicating reduced disease progression. Neurological improvements seen in the pivotal study's primary SARA endpoint were maintained long‐term. The drug remained well‐tolerated, with no treatment‐related AEs reported [117].

#### 7.2.4 Arimoclomol

Arimoclomol is a small‐molecule drug classified as a heat shock protein co‐inducer, targeting HSP70, approved by the FDA in combination with miglustat for the treatment of NPC [19].

##### 7.2.4.1 Arimoclomol Start Criteria

**Statement #17**: All patients with a confirmed diagnosis of NPC and neurological symptoms using background miglustat treatment should be considered for arimoclomol therapy.

- *Strength of recommendation: 2*
- *Level of evidence: B*
- *Expert's opinion: completely agree (37.93%), mostly agree (37.93%), partially agree (20.69%), partially disagree (3.45%), mostly disagree (0.00%), and completely disagree (0.00%)*.

##### 7.2.4.2 Pivotal Clinical Trial Data

A 12‐month multinational phase II/III trial demonstrated that arimoclomol in combination with miglustat significantly slowed disease progression versus placebo in NPC patients aged 2–18 years [19]. Seventy‐eight percent of the patients in this study had previously received miglustat and continued its use throughout the trial.

At 12 months, the mean (±SD) change from baseline in the 5‐domain NPC‐CSS total score was 0.76 with arimoclomol, compared to 2.15 in the placebo group (mean difference, −1.40 points; 95% CI, −2.76, −0.03; *p* = 0.046). Patients who received arimoclomol plus miglustat showed a mean change of −0.06 pts. on the 5‐domain NPC‐CSS, versus a mean change of 4.2 pts. for those patients on arimoclomol alone, indicating that arimoclomol treatment is not effective without miglustat. Adverse events occurred in 30/34 (88.2%) arimoclomol‐treated patients versus 12/16 (75.0%) on placebo [19]. Serious adverse events were less frequent with arimoclomol (5/34; 14.7%) than with placebo (5/16; 31.3%). Treatment‐related serious adverse events (*n* = 2) were urticaria and angioedema [19].

##### 7.2.4.3 Long‐Term Data

Long‐term data from an OLE and Early Access Program support the efficacy and safety of arimoclomol in combination with miglustat in NPC. The 5‐domain NPC‐CSS generally continued to increase during the OLE phase, with mean increases of 3.2 over 48 months, corresponding to annual progression rates of 0.8, lower than the 1.5‐point annual progression rate reported for patients in the natural history, and aligned with the previously reported progression rate for the arimoclomol group in the pivotal phase study [19, 57].

### 7.3 Combination Therapy

**Statement #18**: Combination treatment should be considered for all patients with a confirmed diagnosis of NPC.

- *Strength of recommendation: 2*
- *Level of evidence: C*
- *Expert's opinion: completely agree (37.93%), mostly agree (41.38%), partially agree (20.69%), partially disagree (0.00%), mostly disagree (0.00%), and completely disagree (0.00%)*.

No currently available therapies for NPC are curative. However, available treatments have distinct mechanisms of action, making combination therapy an approach that targets multiple aspects of NPC neuropathology [20]. In both arimoclomol and levacetylleucine pivotal clinical trials, more than 75% of study participants were taking miglustat, highlighting the importance and safety of combination therapy [18, 19]. Additionally, predicting patient treatment responses remains challenging, further supporting the potential benefits of a combined therapeutic approach [118]. Despite this, it is important to note that healthcare system resource limitations often restrict access to combination treatments. Improving the accessibility and affordability of novel therapies is crucial for enhancing patient outcomes in NPC [119].

### 7.4 Experimental Therapies

Clinical trials or expanded access programs are ongoing to evaluate the safety and efficacy of intrathecal or intravenous formulations of 2‐hydroxypropyl‐β‐cyclodextrin [120, 121]. Other experimental therapies include the orally administered agents efavirenz, a non‐nucleoside reverse transcriptase antiretroviral inhibitor, and nizubaglustat, a selective dual inhibitor of ceramide glucosyltransferase and non‐lysosomal neutral glucosylceramidase (NLGase) [122, 123]. In addition, several other therapeutic modalities, including gene therapy, antisense oligonucleotide (ASO) therapy for patients with specific targetable mutations, and CRISPR/Cas9 technology in animal studies, are underway with positive preliminary findings [21, 124].

### 7.5 Future Research

The INPDR is a disease‐specific registry that has been instrumental in advancing NPC research and understanding. Future research priorities must focus on identifying the optimal timing for individual therapies during disease progression and understanding the most effective treatment combinations for various NPC patient subtypes. Observational data captured through INPDR may provide greater clarity on the efficacy of combination therapy.

## 8 Follow‐Up, Transition, Advanced Care Planning and Genetic Issues

### 8.1 Follow‐Up

**Statement #19**: NPC is a progressive condition, and patients require regular follow‐up to monitor disease severity, treatment response, and recurrence. Treatment goals should be established at diagnosis and reviewed regularly, aimed at improving or maintaining the physical and psychosocial wellbeing of individuals with NPC and their families.

- *Strength of recommendation: 1*
- *Level of evidence: B*
- *Expert's opinion: completely agree (100.00%), mostly agree (0.00%), partially agree (0.00%), partially disagree (0.00%), mostly disagree (0.00%), and completely disagree (0.00%)*.

### 8.2 Transition

**Statement #20**: Most children with late infantile and juvenile onset NPC are expected to reach adulthood with complex medical and psychosocial needs. The transition from pediatric to adult services should begin early and requires appropriate services in the community to provide a seamless transition from childhood to adult life. Individuals with NPC may benefit from a detailed assessment identifying barriers to independence.

- *Strength of recommendation: 1*
- *Level of evidence: B*
- *Expert's opinion: completely agree (89.66%), mostly agree (6.90%), partially agree (3.45%), partially disagree (0.00%), mostly disagree (0.00%), and completely disagree (0.00%)*.

### 8.3 Advance Care Planning

**Statement #21**: Specialist center care providers, family physicians/pediatricians, and local palliative care services should develop close working links to support individuals and families with NPC throughout their lifespan. Possible approaches include: (a) advance care planning with regular updating, (b) proper flow of communication and information for patients and their families, and (c) a designated point of contact for each stage in their care pathway. An individual identified as being near the end of life may benefit from ongoing access to palliative care services, including for symptom control, respite, and psychological and spiritual support.

- *Strength of recommendation: 1*
- *Level of evidence: B*
- *Expert's opinion: completely agree (100.00%), mostly agree (0.00%), partially agree (0.00%), partially disagree (0.00%), mostly disagree (0.00%), and completely disagree (0.00%)*.

### 8.4 Genetic Testing

**Statement #22**: Requests for NPC pre‐symptomatic genetic testing are best managed on a case‐by‐case basis. Pre‐symptomatic testing in minors is not permitted in some jurisdictions and, in any case, the risks and benefits from the perspectives of both the child and parents should be carefully discussed in the context of formal counseling from a suitably qualified individual. All individuals with a genetic diagnosis identified pre‐symptomatically should be referred to specialist centers for surveillance and early detection of neurological manifestations.

- *Strength of recommendation: 2*
- *Level of evidence: B*
- *Expert's opinion: completely agree (86.21%), mostly agree (10.34%), partially agree (3.45%), partially disagree (0.00%), mostly disagree (0.00%), and completely disagree (0.00%)*.

**Statement #23**: Prenatal testing and reproductive options for NPC should be offered to all at‐risk couples; this requires careful counseling by clinical geneticists/genetic counselors and NPC specialists. Molecular genetic analysis of chorionic villus samples is the strategy of choice for prenatal diagnosis, based on pathogenic variants identified in the family, as well as preimplantation genetic testing.

- *Strength of recommendation: 2*
- *Level of evidence: B*
- *Expert's opinion: completely agree (62.07%), mostly agree (34.48%), partially agree (3.45%), partially disagree (0.00%), mostly disagree (0.00%), and completely disagree (0.00%)*.

## 9 Conclusion

Considering the emergence of novel research findings and advances in the diagnosis and treatment of NPC, the development and dissemination of the 2025 consensus clinical management guidelines are both timely and critical in providing up‐to‐date, evidence‐based care for children and adults with NPC globally. The rarity and variability of the disease underscore gaps in the literature that challenge the process of defining a standard of NPC care. Nevertheless, the guidelines presented in this paper have been updated using the best available evidence, alongside the expertise and experience of the GDG members. Overall, the current guidelines offer a standardized approach to support optimal international management of NPC for all affected by this ultra‐rare, life‐limiting disorder.

## Author Contributions

All authors contributed to the guideline's development process of planning, writing, and revising the manuscript. All authors read and approved the final manuscript.

## Funding

This work was supported by the International Niemann‐Pick Disease Alliance.

## Ethics Statement

The authors have nothing to report.

## Consent

The authors have nothing to report.

## Conflicts of Interest

See Supporting Information for conflicts of interest statement.

## Supporting information


**Data S1:** Supporting Information.

## Data Availability

Data sharing not applicable to this article as no datasets were generated or analyzed during the current study.
