# Supplementary material for: 2025 Consensus Clinical Management Guidelines for Niemann‐Pick Disease Type C
Source: J Inherit Metab Dis. 2026 Apr 26;49:e70185. doi: 10.1002/jimd.70185 (PMC13110917; doi:10.1002/jimd.70185)
Supplement: Supplementary file 1 — Data S1: Supporting Information. [file JIMD-49-0-s001.docx]

**Supplementary material for 2025 consensus clinical management guidelines for Niemann-Pick disease type C**

**Conflicts of Interest:**

F.D.P. reports research support, from the NICHD Intramural Research program and Cooperative Research and Development Agreements (CRADAs) with Mandos Health and Scenic Biotech, and grants from the Ara Parseghian Medical Research Fund, SOAR-NPC, Firefly Fund, and Together Strong Foundation; patents related to NPC biomarkers; service on data safety monitoring boards and/or advisory boards for Screen Plus, the Ara Parseghian Medical Research Fund, and the National Niemann-Pick Disease Foundation; and travel/meeting support from the Ara Parseghian Medical Research Fund and the National Niemann-Pick Disease Foundation.

T.B.E. reports research support from Baasch-Medicus Stiftung and the University of Bern; consulting fees from Azafaros and Zevra Therapeutics; and service on data safety monitoring boards and/or advisory boards for AstraZeneca and Scenic Bio.

U.R. reports institutional research grants from Amicus, Chiesi, Denali, IntraBio, and Takeda; and honoraria for advisory boards and/or lecture fees from Amicus, Chiesi, Sanofi, and Takeda.

C.H. reports advisory board roles with Zevra Therapeutics and IntraBio; and travel/meeting support from Cyclo Therapeutics.

B.H. reports personal fees for the 5D-NPCCSS study (Rush University, 2025); advisory services for Zevra Therapeutics; a fiduciary/leadership role with Scenic Biotech (payment recorded 22-Mar-2025); and institutional payments as principal investigator for an Azafaros clinical study.

J.H. reports travel/meeting support from Sanofi and Mandos Health, and service on an advisory board for Sanofi.

J.M. is a Trustee of Niemann-Pick UK (NPUK) (unpaid) and reports that the International Niemann-Pick Disease Alliance (INPDA) funded the coordination of the present manuscript (work performed by 67health with Synthesis Health medical writers) and travel/meeting support from INPDA.

H.A. reports honoraria/fees from Takeda, BioMarin, Sanofi, Ultragenyx, Johnson & Johnson, Azafaros, and Innovative Medicines; travel/meeting support from Takeda and BioMarin; and service on data safety monitoring boards and/or advisory boards for Amicus Therapeutics.

F.D. reports personal consulting fees and payment or honoraria from Sanofi Genzyme, Takeda, and Actelion Pharmaceutical; travel/meeting support from Sanofi Genzyme and Takeda; and service on data safety monitoring boards and/or advisory boards for Sanofi Genzyme.

F.E. reports service on a data safety monitoring board for a clinical trial sponsored by JCR; and roles as principal investigator on clinical trials sponsored by Sanofi, JCR, Denali, Cyclo Therapeutics, PTC, Gemmabio, Novo Nordisk, Amgen, and Amicus.

P.G. reports institutional consulting fees from Bloomsbury Genetic Therapies and institutional research support from Bloomsbury Genetic Therapies toward clinical-trial readiness for gene therapy in Niemann–Pick disease type C (2022–2024).

J.B.G. reports an institutional metabolic consultancy contract with the Texas DSHS Newborn Screening Program and institutional support from Mirum Pharmaceuticals for the RESTORE study; inherited stock and warrants in Occidental Petroleum (July 2024); and receipt of medical-writing services provided by Mirum Pharmaceuticals (no goods/funds to the investigator or institution).

F.P. reports a service contract with IntraBio; a grant from the Ara Parseghian Medical Research Fund, and a joint grant from Orphazyme; personal consulting fees from IntraBio; historical honoraria and travel/meeting support from Actelion Pharmaceutical; stock as an academic co-founder of IntraBio; a patent related to acetyl-DL-leucine (IntraBio); and a fiduciary/leadership role as a Trustee of Niemann-Pick UK (NPUK).

K.J. reports fees for medical-expert engagements with Zevra Therapeutics (U.S. FDA advisory committee, Aug 2024), Cyclo Therapeutics (spring 2025), and Clinical Outcomes Solutions (June 2025).

N.K. reports speaking honoraria from Salveo, Makpharm, and Remedica; and travel/meeting support from Salveo and INOPharm.

A.L. reports funding for research, travel, or advisory board consultations from Sanofi Genzyme, Ultragenyx, Alexion, PTC Therapeutics, Amicus, Takeda, BioMarin, Medison, Horizon, and Idorsia.

Y.N. reports a personal fee/honorarium from Zevra Therapeutics.

S.A.S. reports an institutional grant from the Ara Parseghian Medical Research Fund; personal consulting fees from Azafaros; and a fiduciary/leadership role in the Movement Disorders Society.

S.J. reports consulting fees from Orchard Therapeutics, Novartis, Eli Lilly, and Spruce; and travel/meeting support from Sanofi.

E.M. reports research grants from Sanofi, Zevra/Orphazyme, Freeline Therapeutics, Cyclo Therapeutics, Idorsia, JCR, Denali, Amicus, IntraBio, and Prevail; advisory/consulting roles with Sanofi, Zevra/Orphazyme, Freeline Therapeutics, Cyclo Therapeutics, Idorsia, JCR, Denali, Amicus, and Prevail; and payment/honoraria from Sanofi, Zevra/Orphazyme, Cyclo Therapeutics, Alexion, Genorph, Amicus, and Prevail.

M.W. reports funding from IntraBio and Cyclo Therapeutics to undertake a clinical trial.

O.G.A. reports a research grant/contract with Cyclo Therapeutics.

C.D. reports participation in industry-supported meetings and advisory boards without accepting personal honoraria or support.

S.C. is a Trustee of the INPDA. Within the past 36 months, INPDA has received unrestricted educational grants from Azafaros B.V., IntraBio, Cyclo Therapeutics, Mandos Health and Zevra Therapeutics in support of patient-advocacy activities. No personal payments related to this work were received by the author.

T.M. is a Trustee of the INPDA and a paid employee of NPUK. Within the past 36 months, INPDA has received unrestricted educational grants from Azafaros B.V., IntraBio, Cyclo Therapeutics, Mandos Health and Zevra Therapeutics in support of patient-advocacy activities. No personal payments related to this work were received by the author.

E.B.K. reports institutional funding from Zevra Therapeutics for an expanded-access program; institutional payments for consulting provided to Zevra Therapeutics and IntraBio; and meeting registration support from Zevra Therapeutics.

M.C.P. reports grant support to Mayo Clinic through 31-Dec-2024 from Azafaros, Orphazyme, KemPharm, Zevra Therapeutics, Glycomine, Shire-Takeda, and Maggie’s Pearl; an editor’s stipend from SAGE Publishing through 31-Dec-2024; employment with IntraBio since 1-Jan-2025; personal royalties from Wolters Kluwer; personal consulting fees from Azafaros and IntraBio; travel support from SSIEM for editors’ meetings; unpaid service on the NNPDF and APMRF scientific advisory boards and as INPDR Scientific Advisory Committee (SAC) Chair (through 31-Dec-2024); and personal stock/stock options in IntraBio.

The other authors declare no conflicts of interest.
